# *N*,*N*,*N*′,*N*′-Tetra­methyl-*N*′′-[2-(*N*′,*N*′,*N*′′,*N*′′-tetra­methyl­guanidino)eth­yl]guanidine

**DOI:** 10.1107/S1600536812027079

**Published:** 2012-06-20

**Authors:** Ioannis Tiritiris, Willi Kantlehner

**Affiliations:** a Institut für Organische Chemie, Universität Stuttgart, Pfaffenwaldring 55, 70569 Stuttgart, Germany; b Fakultät Chemie/Organische Chemie, Hochschule Aalen, Beethovenstrasse 1, D-73430 Aalen, Germany

## Abstract

The title compound, C_12_H_28_N_6_, is located about an inversion center situated at the center of the —CH_2_—CH_2_— bond. The C—N bond lengths are 1.285 (2), 1.384 (2) and 1.395 (1) Å, indicating double- and single-bond character. The N—C—N angles are 114.1 (1), 119.3 (1) and 126.5 (1)°, showing a deviation of both CN_3_ planes from an ideal trigonal–planar geometry.

## Related literature

For the crystal structure of *N*,*N*,*N*′,*N*′-tetra­methyl­chloro­formamidinium-chloride, see: Tiritiris & Kantlehner (2008 ▶). For the synthesis of *N*,*N*,*N*′,*N*′-tetra­methyl-*N*′′-[2-(*N*′,*N*′,*N*′′,*N*′′-tetra­methyl­guanidino)-eth­yl]-guanidine and the crystal structure of the corresponding diprotonated bis­guanidinium dichloride salt, see: Wittmann *et al.* (2000 ▶). For the synthesis and characterization of bis­guanidine–copper complexes, see: Bienemann *et al.* (2010 ▶). 
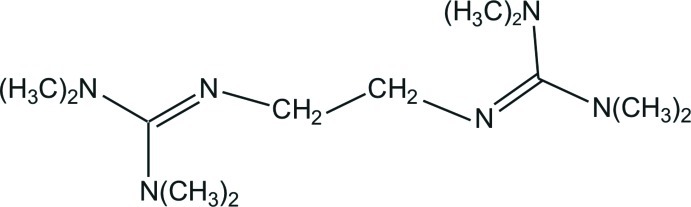


## Experimental

### 

#### Crystal data

C_12_H_28_N_6_
*M* *_r_* = 256.40
Monoclinic, 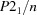

*a* = 8.4189 (6) Å
*b* = 8.5894 (6) Å
*c* = 11.0089 (8) Å
β = 106.858 (5)°
*V* = 761.88 (10) Å^3^
*Z* = 2
Mo *K*α radiation
μ = 0.07 mm^−1^
*T* = 293 K
0.22 × 0.18 × 0.15 mm

#### Data collection

Bruker–Nonius KappaCCD diffractometer
7247 measured reflections
1835 independent reflections
1120 reflections with *I* > 2σ(*I*)
*R*_int_ = 0.043

#### Refinement

*R*[*F* ^2^ > 2σ(*F* ^2^)] = 0.036
*wR*(*F* ^2^) = 0.096
*S* = 0.86
1835 reflections
87 parameters
H-atom parameters constrained
Δρ_max_ = 0.11 e Å^−3^
Δρ_min_ = −0.13 e Å^−3^

### 

Data collection: *COLLECT* (Hooft, 2004 ▶); cell refinement: *SCALEPACK* (Otwinowski & Minor, 1997 ▶); data reduction: *SCALEPACK*; program(s) used to solve structure: *SHELXS97* (Sheldrick, 2008 ▶); program(s) used to refine structure: *SHELXL97* (Sheldrick, 2008 ▶); molecular graphics: *DIAMOND* (Brandenburg & Putz, 2005 ▶); software used to prepare material for publication: *SHELXL97*.

## Supplementary Material

Crystal structure: contains datablock(s) I, global. DOI: 10.1107/S1600536812027079/kp2426sup1.cif

Structure factors: contains datablock(s) I. DOI: 10.1107/S1600536812027079/kp2426Isup2.hkl

Additional supplementary materials:  crystallographic information; 3D view; checkCIF report

## Figures and Tables

**Fig. 1.**
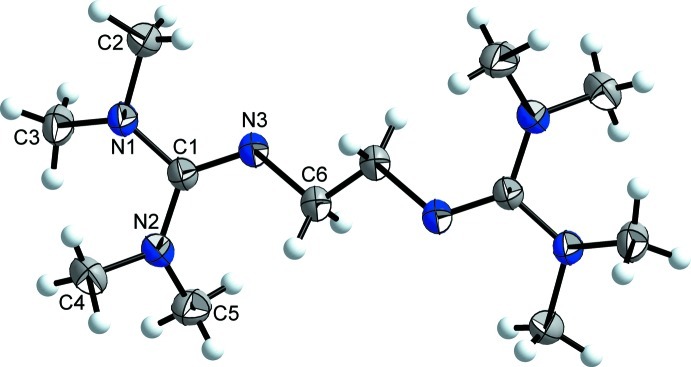
The structure of the title compound with atom labels and 50% probability displacement ellipsoids.

## supplementary crystallographic information

### Comment

The synthesis of *N*,*N*,*N*',*N*'-tetramethyl-*N*''- [2-(*N*',*N*',*N*'',*N*''-tetramethylguanidino)-ethyl]- guanidine is well known in literature (Wittmann *et al.*, 2000). The compound was used as a nitrogen donor ligand in reactions with copper halogenides (CuI or CuCl_2_), to give mono- or bis-chelate complexes (Bienemann *et al.*, 2010). However, the crystal structure of the free guanidine base was previously unknown. According to the structure analysis, the C1–N3 bond in the bisguanidine is 1.285 (2) Å, indicating double bond character. The bond lengths C1–N2 = 1.384 (2) Å and C1–N1 = 1.395 (1) Å are elongated and characteristic for a C–N imine single bond. The N–C1–N angles are 114.1 (1)° (N1–C1–N2), 126.5 (1)° (N2–C1–N3) and 119.3 (1)° (N1–C1–N3), showing a deviation of both CN_3_ planes from an ideal trigonal planar geometry (Fig. 1). The dihedral angle N3—C6—C6^i^—N3^i^ is 180.00 (9). The bonds between the N atoms and the terminal *C*-methyl groups, all have values close to a typical single bond (1.442 (2)–1.459 (1) Å). This is completely different compared with the geometrical parameters from the crystal structure analysis of the corresponding diprotonated bisguanidinium dichloride salt (Wittmann *et al.*, 2000). Here, the C–N bond lengths of the CN_3_ units are in a range between 1.336 (2) and 1.342 (2) Å, the N–C–N angles are 119.5 (1), 120.1 (1) and 120.4 (1)°, indicating also delocalization of the positive charges on both CN_3_ planes. The crystal packing in the here presented title compound is through van der Waals interactions, only.

### Experimental

Two equivalents of *N*,*N*,*N*',*N*'-tetramethylchloroformamidinium-chloride (Tiritiris & Kantlehner, 2008) were reacted with one equivalent of ethane-1,2-diamine in acetonitrile in the presence of triethylamine at 273 K. The obtained protonated bisguanidinium dichloride salt was reacted in a next step with an aqueous sodium hydroxide solution at 273 K. After extraction of the bisguanidine with diethyl ether from the water phase, the solvent was evaporated and the title compound was isolated in form of a colourless solid. Single crystals have been obtained by recrystallization from a saturated acetonitrile solution.

### Refinement

The hydrogen atoms of the methyl groups were allowed to rotate with a fixed angle around the C–N bond to best fit the experimental electron density, with *U*(H) set to 1.5 *U*_eq_(C) and d(C—H) = 0.96 Å. The remaining H atoms were placed in calculated positions with d(C—H) = 0.97 Å. They were included in the refinement in the riding model approximation, with *U*(H) set to 1.2 *U*_eq_(C).

### Crystal data

**Table:**

C_12_H_28_N_6_	*F*(000) = 284
*M**_r_* = 256.40	*D*_x_ = 1.118 Mg m^−^^3^
Monoclinic, *P*2_1_/*n*	Mo *K*α radiation, λ = 0.71073 Å
Hall symbol: -P 2yn	Cell parameters from 7247 reflections
*a* = 8.4189 (6) Å	θ = 2.7–28.1°
*b* = 8.5894 (6) Å	µ = 0.07 mm^−^^1^
*c* = 11.0089 (8) Å	*T* = 293 K
β = 106.858 (5)°	Polyhedral, colourless
*V* = 761.88 (10) Å^3^	0.22 × 0.18 × 0.15 mm
*Z* = 2	

### Data collection

**Table:**

Bruker–Nonius KappaCCD diffractometer	1120 reflections with *I* > 2σ(*I*)
Radiation source: sealed tube	*R*_int_ = 0.043
Graphite monochromator	θ_max_ = 28.1°, θ_min_ = 2.7°
φ scans, and ω scans	*h* = −11→11
7247 measured reflections	*k* = −11→11
1835 independent reflections	*l* = −14→14

### Refinement

**Table:**

Refinement on *F*^2^	Secondary atom site location: difference Fourier map
Least-squares matrix: full	Hydrogen site location: difference Fourier map
*R*[*F*^2^ > 2σ(*F*^2^)] = 0.036	H-atom parameters constrained
*wR*(*F*^2^) = 0.096	*w* = 1/[σ^2^(*F*_o_^2^) + (0.059*P*)^2^] where *P* = (*F*_o_^2^ + 2*F*_c_^2^)/3
*S* = 0.86	(Δ/σ)_max_ < 0.001
1835 reflections	Δρ_max_ = 0.11 e Å^−^^3^
87 parameters	Δρ_min_ = −0.13 e Å^−^^3^
0 restraints	Extinction correction: *SHELXL97* (Sheldrick, 2008), Fc^*^=kFc[1+0.001xFc^2^λ^3^/sin(2θ)]^-1/4^
Primary atom site location: structure-invariant direct methods	Extinction coefficient: 0.57 (2)

### Special details

**Table:**

Geometry. All e.s.d.'s (except the e.s.d. in the dihedral angle between two l.s. planes) are estimated using the full covariance matrix. The cell e.s.d.'s are taken into account individually in the estimation of e.s.d.'s in distances, angles and torsion angles; correlations between e.s.d.'s in cell parameters are only used when they are defined by crystal symmetry. An approximate (isotropic) treatment of cell e.s.d.'s is used for estimating e.s.d.'s involving l.s. planes.
Refinement. Refinement of *F*^2^ against ALL reflections. The weighted *R*-factor *wR* and goodness of fit *S* are based on *F*^2^, conventional *R*-factors *R* are based on *F*, with *F* set to zero for negative *F*^2^. The threshold expression of *F*^2^ > σ(*F*^2^) is used only for calculating *R*-factors(gt) *etc*. and is not relevant to the choice of reflections for refinement. *R*-factors based on *F*^2^ are statistically about twice as large as those based on *F*, and *R*- factors based on ALL data will be even larger.

### Fractional atomic coordinates and isotropic or equivalent isotropic displacement parameters (Å^2^)

**Table:**

	*x*	*y*	*z*	*U*_iso_*/*U*_eq_	
N1	−0.04772 (12)	0.18588 (12)	0.32603 (9)	0.0485 (3)	
N2	0.20678 (12)	0.08543 (13)	0.31899 (10)	0.0556 (3)	
N3	−0.01179 (12)	0.13388 (11)	0.12955 (9)	0.0484 (3)	
C1	0.05014 (13)	0.13573 (13)	0.25085 (10)	0.0434 (3)	
C2	−0.22265 (16)	0.20743 (17)	0.26375 (13)	0.0607 (4)	
H2A	−0.2392	0.3035	0.2173	0.091*	
H2B	−0.2819	0.2105	0.3262	0.091*	
H2C	−0.2631	0.1226	0.2064	0.091*	
C3	0.01949 (17)	0.29870 (15)	0.42638 (12)	0.0571 (3)	
H3A	0.1384	0.2914	0.4535	0.086*	
H3B	−0.0228	0.2775	0.4968	0.086*	
H3C	−0.0128	0.4017	0.3950	0.086*	
C4	0.24173 (19)	0.01111 (18)	0.44203 (13)	0.0688 (4)	
H4A	0.2972	0.0835	0.5069	0.103*	
H4B	0.3116	−0.0778	0.4444	0.103*	
H4C	0.1396	−0.0216	0.4563	0.103*	
C5	0.35164 (16)	0.12927 (18)	0.28218 (14)	0.0664 (4)	
H5A	0.3186	0.1914	0.2065	0.100*	
H5B	0.4073	0.0373	0.2663	0.100*	
H5C	0.4254	0.1883	0.3492	0.100*	
C6	0.06621 (15)	0.04024 (13)	0.05215 (10)	0.0488 (3)	
H6A	0.1385	−0.0369	0.1049	0.059*	
H6B	0.1331	0.1064	0.0150	0.059*	

### Atomic displacement parameters (Å^2^)

**Table:**

	*U*^11^	*U*^22^	*U*^33^	*U*^12^	*U*^13^	*U*^23^
N1	0.0438 (5)	0.0632 (6)	0.0376 (5)	−0.0024 (4)	0.0103 (4)	−0.0085 (4)
N2	0.0442 (5)	0.0785 (7)	0.0409 (6)	0.0042 (5)	0.0074 (4)	0.0036 (5)
N3	0.0528 (6)	0.0556 (6)	0.0351 (5)	0.0067 (4)	0.0099 (4)	−0.0028 (4)
C1	0.0434 (6)	0.0495 (6)	0.0357 (6)	−0.0011 (4)	0.0090 (5)	−0.0028 (4)
C2	0.0463 (7)	0.0865 (9)	0.0482 (7)	0.0030 (6)	0.0121 (6)	−0.0075 (6)
C3	0.0635 (8)	0.0643 (8)	0.0439 (7)	−0.0071 (6)	0.0162 (6)	−0.0122 (5)
C4	0.0686 (9)	0.0823 (9)	0.0476 (8)	0.0144 (7)	0.0043 (7)	0.0078 (7)
C5	0.0458 (7)	0.0868 (10)	0.0662 (9)	−0.0047 (6)	0.0155 (7)	−0.0165 (7)
C6	0.0518 (7)	0.0576 (7)	0.0361 (6)	0.0056 (5)	0.0115 (5)	−0.0016 (5)

### Geometric parameters (Å, º)

**Table:**

N1—C1	1.3945 (14)	C3—H3B	0.9600
N1—C2	1.4449 (16)	C3—H3C	0.9600
N1—C3	1.4550 (15)	C4—H4A	0.9600
N2—C1	1.3837 (15)	C4—H4B	0.9600
N2—C5	1.4422 (16)	C4—H4C	0.9600
N2—C4	1.4483 (17)	C5—H5A	0.9600
N3—C1	1.2852 (15)	C5—H5B	0.9600
N3—C6	1.4589 (13)	C5—H5C	0.9600
C2—H2A	0.9600	C6—C6^i^	1.516 (2)
C2—H2B	0.9600	C6—H6A	0.9700
C2—H2C	0.9600	C6—H6B	0.9700
C3—H3A	0.9600		
			
C1—N1—C2	117.02 (10)	H3A—C3—H3C	109.5
C1—N1—C3	119.32 (9)	H3B—C3—H3C	109.5
C2—N1—C3	113.16 (10)	N2—C4—H4A	109.5
C1—N2—C5	121.12 (11)	N2—C4—H4B	109.5
C1—N2—C4	123.22 (10)	H4A—C4—H4B	109.5
C5—N2—C4	114.76 (11)	N2—C4—H4C	109.5
C1—N3—C6	119.87 (10)	H4A—C4—H4C	109.5
N3—C1—N2	126.52 (10)	H4B—C4—H4C	109.5
N3—C1—N1	119.28 (11)	N2—C5—H5A	109.5
N2—C1—N1	114.14 (10)	N2—C5—H5B	109.5
N1—C2—H2A	109.5	H5A—C5—H5B	109.5
N1—C2—H2B	109.5	N2—C5—H5C	109.5
H2A—C2—H2B	109.5	H5A—C5—H5C	109.5
N1—C2—H2C	109.5	H5B—C5—H5C	109.5
H2A—C2—H2C	109.5	N3—C6—C6^i^	109.70 (12)
H2B—C2—H2C	109.5	N3—C6—H6A	109.7
N1—C3—H3A	109.5	C6^i^—C6—H6A	109.7
N1—C3—H3B	109.5	N3—C6—H6B	109.7
H3A—C3—H3B	109.5	C6^i^—C6—H6B	109.7
N1—C3—H3C	109.5	H6A—C6—H6B	108.2
			
C6—N3—C1—N2	15.52 (18)	C2—N1—C1—N3	10.45 (16)
C6—N3—C1—N1	−161.37 (10)	C3—N1—C1—N3	−132.01 (12)
C5—N2—C1—N3	46.34 (18)	C2—N1—C1—N2	−166.81 (11)
C4—N2—C1—N3	−145.11 (14)	C3—N1—C1—N2	50.74 (15)
C5—N2—C1—N1	−136.65 (12)	C1—N3—C6—C6^i^	138.63 (13)
C4—N2—C1—N1	31.90 (17)		

Symmetry code: (i) −*x*, −*y*, −*z*.
